# Complex rearranged small supernumerary marker chromosomes (sSMC), three new cases; evidence for an underestimated entity?

**DOI:** 10.1186/1755-8166-1-6

**Published:** 2008-04-15

**Authors:** Vladimir Trifonov, Simon Fluri, Franz Binkert, Adayapalam Nandini, Jasen Anderson, Laura Rodriguez, Madeleine Gross, Nadezda Kosyakova, Hasmik Mkrtchyan, Elisabeth Ewers, Daniela Reich, Anja Weise, Thomas Liehr

**Affiliations:** 1 Department of Clinical Veterinary Medicine, Madingley Road, Cambridge, CB3 OES, UK; 2 Institut für Humangenetik und Anthropologie, Kollegiengasse 10, D-07743 Jena, Germany; 3 Universitätskinderklinik, Inselspital, CH-3010 Bern, Switzerland; 4 MCL Medizinische Laboratorien, Freiburgstr 634, 3127 Niederwangen, Switzerland; 5 Department of Cytogenetics, Queensland Health Pathology Services, Herston QLD 4029, Queensland, Australia; 6 Department of Cytogenetics, Sullivan Nicolaides Pathology, Taringa QLD, Australia; 7 Estudio Colaborativo Español de Malformaciones Congénitas (ECEMC) del Centro de Investigación sobre Anomalías Congénitas (CIAC), Instituto de Salud Carlos III, Ministerio de Sanidad y Consumo, Madrid, Spain

## Abstract

**Background:**

Small supernumerary marker chromosomes (sSMC) are present ~2.6 × 10^6 ^human worldwide. sSMC are a heterogeneous group of derivative chromosomes concerning their clinical consequences as well as their chromosomal origin and shape. Besides the sSMC present in Emanuel syndrome, i.e. der(22)t(11;22)(q23;q11), only few so-called complex sSMC are reported.

**Results:**

Here we report three new cases of unique complex sSMC. One was a *de novo *case with a dic(13 or 21;22) and two were maternally derived: a der(18)t(8;18) and a der(13 or 21)t(13 or 21;18). Thus, in summary, now 22 cases of unique complex sSMC are available in the literature. However, this special kind of sSMC might be under-diagnosed among sSMC-carriers.

**Conclusion:**

More comprehensive characterization of sSMC and approaches like reverse fluorescence in situ hybridization (FISH) or array based comparative genomic hybridization (array-CGH) might identify them to be more frequent than only ~0.9% among all sSMC.

## Background

Small supernumerary marker chromosomes (sSMC) are a major problem in cytogenetic diagnostics and genetic counseling. sSMC are structurally abnormal chromosomes that cannot be identified or characterized unambiguously by conventional banding cytogenetics alone, and are generally about the size of or smaller than a chromosome 20 in the same metaphase spread. Molecular cytogenetic techniques are necessary for comprehensive sSMC characterization [1]. Cases with a *de novo *sSMC, particularly those that are prenatally ascertained, are not easy to correlate with a clinical outcome [2]. It has been established that substantial parts of sSMC lead to four specific syndromes, i.e. Pallister-Killian [= i(12p)], isochromosome 18p [i(18p)], cat-eye [i(22p~q)], and Emanuel or derivative chromosome 22 [der(22)t(11;22)] syndromes [1]. Moreover, for the remaining ones, recently a first step towards a genotype-phenotype correlation was reported [2]. In general, the risk for an abnormal phenotype in prenatally ascertained *de novo *cases with sSMC is considered to be ~13% [3]. This has been refined to 7% (for sSMC from chromosome 13, 14, 21 or 22) and 28% (for all non-acrocentric autosomes) [4] and has now been suggested to be 30% [5]. Also generally speaking, sSMC transmitted by normal sSMC carriers to their progeny are not correlated with clinical problems [6], although exceptions have been described [7].

One of the smallest subgroup of sSMC is constituted by the so-called complex marker chromosomes. 'Complex' are such sSMC which consist of chromosomal material derived from more than one chromosome [1]. Thus, besides the aforementioned larger group of Emanuel- or derivative chromosome 22- [der(22)t(11;22)-] syndrome cases, up to now only 19 unique complex sSMC were described (see Tab. 1). Here we report three more such cases of unique complex sSMC and provide a review of the literature. Moreover, it is discussed if this kind of sSMC is under-diagnosed due to lack of appropriate screening techniques.

**Table 1:** Cases with unique complex sSMC reported in the literature.

**Case**	**Case acc. to [10]**	**GTG-karyotype**	**sSMC acc. to FISH**	**abnormal clinical outcome**
de novo				

1	07-U-1	47,XX,+mar [100%]	der(7)t(X;5;7)(p22.1;q35;p13q21)	+
2	13/21-U27	47,XY,+mar [100%]	der(13 or 21)t(13 or 21;18)(13 or 21pter->13 or 21q11::18p11.21->18pter)	+
3	13/21-U-8	47,XX,+mar [100%]	der(13 or 21)t(13 or 21;18)(q11;p11.2)	?
4	14-O-q11.2/1-1 15-O-q11.1/4-1	47,XY,+mar [100%]	dic(14;15)(14pter->14q11.2::15q11.1->15pter)	-
5	15-CW-3	47,XX,+mar [100%]	der(15)t(15;?)(q24;?)	+
6	15-U-6 22-U-4	47,XY,+mar [100%]	dic(15;22)(q11.1;q22.1)	?
7	15-U-10	47,XY,+mar [100%]	der(15)t(Y;15)(q12;q22)	?
8	17-W-p13.3/1-1	47,XYqs,+mar [100%]	der(17)t(17;acro)(q11;p11.2)	+
9	22-U-18	47,XY,+mar [100%]	der(22)t(12;22)(p12;q11.2-12)	+
10*	22-Wces-5-101	47,XX,+mar [100%]	dic(13 or 21;22)(13 or 21pter->13 or21q11::22q11.1~11.2->22q11.21~11.22::22q11.21~11.22->22pter)	+

Unclear origin				

11	15-CO-1 0Y-CO-2	47,XX,+mar [100%]	dic(Y;15) presence of 2 alpha-cepY and cep15 signals; PCR prove of Yq11 euchromatic region (AZF1); absence of SRY region	-
12	21-O-q11.1/1-1 22-O-q11.1/3-1	46,t_ROB_(21;22),+mar [100%]	der(21)t(21;22)(q11.1;p11.2)	-

sSMC from mother				

13*	18-U-10	47,XY,+mar [100%]	der(18)t(8;18)(8p23.2~23.1;18q11.1)	+
14	13/21-O-q10/4-1 14-O-q10/2-1	47,XX,+mar [87%]/46,XX [13%]	dic(13 or 21;14)(q10;q10)	-
15	13/21-O-q10/5-1 15-O-q10/4-1	47,XX,+mar [100%]	dic(13 or 21;15)(q10;q10)	-
16*	13/21-U-28	47,XX,+mar [100%]	der(13 or 21)t(13 or 21;18)(13 or 21pter->13 or 21q11::18p11.21->18pter)	+

Parental balanced translocation				

17	12-U-6	47,+mar [100%]	der(12)t(4;12)(p16;q11) mat	+
18	13-U-8	47,XY,+mar [100%]	der(13)t(8;13)(p23.2;q12.2) mat	+
19	15-O-q11.2/5-1	47,XY,+mar [100%]	der(15)t(9;15)(p24;q11.2) mat	-
20	15-U-15	47,XX,+mar [100%]	der(15)t(15;16)(q13;p13.2) mat	+
21	18-CW-2	47,XX,+mar [100%]	der(18)t(18;21 or 22) mat der(18)t(18;21 or 22) pat	+
22	22-U-11	47,XY,+mar [100%]	der(22)t(8;22)(q24.1;q11.2) pat	+

All cases with unique complex sSMC reported in the literature according to [10]; the case numbering scheme is explained also in Ref. 10. GTG-karyotype, sSMC as characterized after FISH and information on the clinical outcome are provided. The three new cases reported here are marked by asterisks.

## Results and discussion

### Case reports

#### Case A (= #13 in Tab. 1)

A 13 months old boy was studied cytogenetically because of failure to thrive and psychomotor development delay. The patient was the product of the fourth pregnancy of a non-consanguineous couple. At birth, the mother was 31 and the father 55 years old. The pregnancy was complicated by hyperemesis gravidarum treated with metoclopramid; additionally, there was a mild exposure to tobacco (3 cigarettes a day) and to alcohol (1 unit per month). Sonography was normal during whole pregnancy. The patient was born by normal vaginal delivery at 41 1/7 weeks of gestation with a birth weight of 2150 g (10^th ^centile), a length of 52 cm (50^th ^to 90^th ^centile) and a head circumference of 34 cm (10^th ^centile). Neonatal adaptation was good with an APGAR-score of 9/10/10 and no apparent congenital abnormalities were noticed. During the first year of live, the patient showed an increasing refusal to eat with insufficient growth (weight -3.6 SDS, length 2.4 SDS, head circumference 3.8 SDS at the age of 13 months). At 13 months, psychomotor development was markedly delayed with a Griffith General Intelligence Quotient of 63. The neurological examination revealed slight muscular hypotonia but otherwise no abnormalities. MRI of the brain was normal. Laboratory analyses were not suggestive for any metabolic disorder.

For family history: the mother was treated for attention-deficit/hyperactivity problems during her adolescence and her IQ was borderline. The father presented no medical problems. The patient's 6 year old brother was born with diaphragmatic hernia and is at present treated for attention-deficit disorder. The 4 years old brother showed a delayed speech development with first words at the age of 3 years but to date his linguistic performance was normal. A fourth child was lost in the 12th week of gestation due to unknown reasons. A neuropsychological development therapy was initiated.

**Figure 1:**
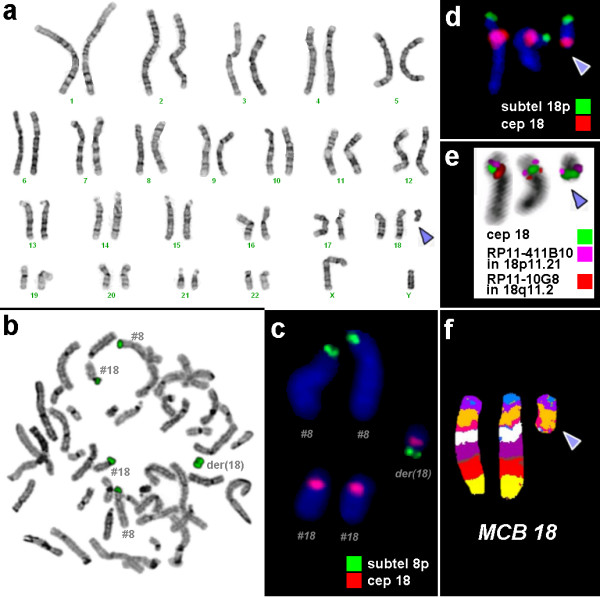
**Cytogenetic and molecular cytogenetic results of case A (= case 13 in Tab.**1**).** The marker is highlighted by a blue arrowhead in a, d, e and f. a) GTG-banding revealed a karyotype 47,XY,+mar in all studied cells. b) Chromosome flow sorting and reverse FISH revealed that the sSMC [= der(18)] consists of chromosome 18p and 8pter material. c) The result of reverse FISH was confirmed by a subtelomeric probe for 8pter (green) and the centromeric probe D18Z1 (= cep 18 – red). d) A subtelomeric probe for 18pter (green) together with D18Z1 (cep 18 – red) indicated that the entire short arm of chromosome 18 was present on the marker chromosome. e) Application of probe D18Z1 with the centromere-near probes for 18p11.21 and 18q11.2 with the showed that obviously no 18q-material was present on the sSMC. f) Multicolor banding (MCB) confirmed that the whole short arm was present three times in this case. There was no hint on additional material of other chromosomal material on the sSMC by this approach.

Banding cytogenetics revealed a karyotype 47,XY,+mar mat [100%]. cenM-FISH uncovered a derivative chromosome 18 (result not shown), which was further characterized to include the entire short arm of chromosome 18. The subtelomeric probe for 18pter (Fig. 1d), the centromere-near probe RP11-411B10 in 18p11.21 and the centromeric probe D18Z1 (Fig. 1e) were present on the sSMC. MCB applying a probe set for chromosome 18 did stain the whole sSMC without leaving any region unstained (Fig. 1f). Surprisingly, flow sorting and reverse FISH of the sSMC revealed the presence of additional material on the derivative chromosome 18, which originated from chromosome 8pter (Fig. 1b). This result was confirmed by a centromeric probe for chromosome 18 applied in combination with a subtelomeric one for chromosome 8pter (Fig. 1c). Thus, a partial trisomy 18p plus 8pter was present in case A and his mother due to an sSMC der(18)t(8;18)(8p23.2~23.1;18q11.1).

#### Case B (= case #16 in Tab. 1)

A newborn male, born at 42 weeks of gestation by normal vaginal delivery, was product of the first pregnancy of a healthy and not consanguineous couple. At birth the mother was 23 years old and the father 38. During whole pregnancy an exposure to tobacco (3 cigarettes a day) was present. Sonography performed in 5^th ^month of pregnancy revealed an artrial septal defect (ASD) and a club foot on the right side. Pregnancy was continued and birth weight was 2,760 g (3^rd ^to 25^th ^centile) with a length 49 cm (25^th ^to 50^th ^centile) and an occipito-frontal circumference (OFC) of 33 cm (25^th ^centile). The prenatally observed findings were confirmed, but no other congenital defects reported. At present the child is two years old, and his development is normal without any delay. The parents are phenotypically normal, even though the mother seems to have a border line IQ.

**Figure 2:**
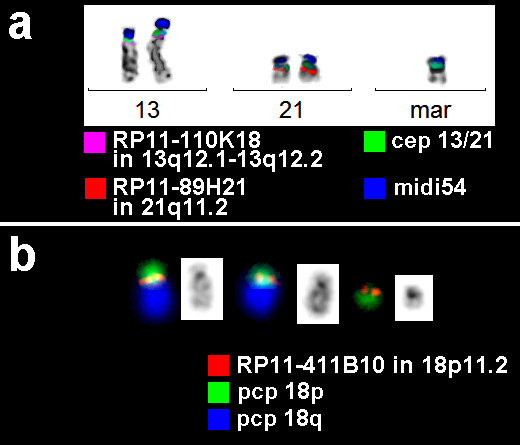
**a) Molecular cytogenetics revealed in case B (= case 16 in Tab. **1**) that the sSMC was derived from chromosome 13 or 21, as the centromeric probe (D13/21Z1 – green) and a probe specific for all acrocentric p-arms (midi54 – blue) were present on the marker.** However, no centromere-near material, neither from chromosome 13 nor 21 was detectable on the marker (pink and red probes). b) Three-color-FISH using partial chromosome painting (pcp) probe for the short (green) and the long arm of chromosome 18 (blue) together with a probe for the centromere-near region of 18p11.2 (red) revealed that the whole short arm was present on the sSMC.

A maternally derived, NOR-positive sSMC was detected in this case in all studied cells. By application of commercially available centromeric probes for the acrocentric chromosomes a derivative chromosome 13 or 21 was characterized. As well-known, centromeres of chromosomes 13 and 21 harbor identical repetitive elements – thus, one cannot decide for a heterochromatic sSMC from which of the both chromosomes the sSMC derives. Centromere-near probes for chromosomes 13 and 21 were not indicative for euchromatic material on the sSMC (Fig. 2a). However, a big part of the sSMC remained unstained by whole chromosome painting probes for chromosome 13 or 21 (results not shown). Thus, glass needle based microdissection of the sSMC followed by reverse FISH were done, which demonstrated that the yet unstained part of the derivative chromosome 13 or 21 was derived from the short arm of chromosome 18 (results not shown). While the centromere-near probe RP11-411B10 in 18p11.21 was present (Fig. 2b), the centromeric probe D18Z1 was absent on the sSMC (results not shown) with the karyotype der(13 or 21)t(13 or 21;18)(13 or 21pter->13 or 21q11::18p11.21->18pter).

#### Case C (= case #10 in Tab. 1)

A 14 month old female with significant developmental delay, cardiac anomalies, preauricular tags, dysmorphism, polyspenia, extrahepatic biliary atresia, intestinal malrotation and hearing loss in right ear was referred to cytogenetic analysis in connection with thrombocytopenia following liver transplant. Clinically a cat eye syndrome was suggested. Bone marrow showed complete replacement with chronic lymphocytic leukemia and marked reduction in erythroid precursors (pure red cell aplasia). Following bone marrow transplant the patient remained in remission. The parents were phenotypically normal.

**Figure 3:**
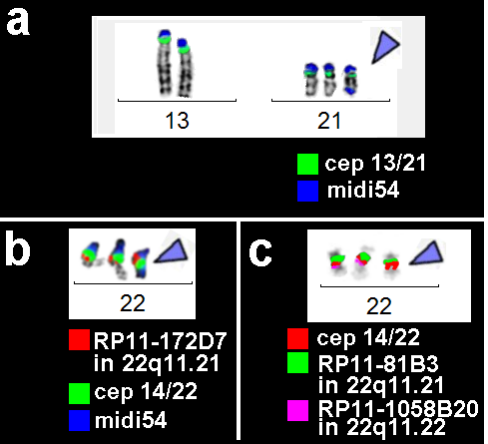
**a) On the sSMC of case C (= case 10 in Tab.**1**– marked by blue arrowhead throughout whole figure) a signal for D13/21Z1 (cep 13/21-green) and two signals for a probe specific for all acrocentric p-arms (midi54 – blue) was obtained.** b) Combining the centromeric probe D14/22Z1 (cep 14/22 – green) with midi54 (blue) and a centromere-near probe in 22q11.21 a tetrasomy of this region was detected. c) The size of the duplication was determined by application of two further probes located in 22q11.21 and 22q11.22.

A *de novo *sSMC was present in all studied cells of this case. The NOR-positive sSMC was initially characterized as a derivative of chromosome 13 or 21 by application of the corresponding commercially available centromeric probes for #13/21 (D13/21Z1) and #15 (D15Z1); these probes were applied, as they were available in the Australian laboratory where the case was detected. As a probe specific for the short arms of all acrocentric chromosomes (midi54) gave two signals on the sSMC (see Fig. 3a) also the probe for the centromeric regions 14 or 22 (D14/22Z1) was applied and gave one signal on the sSMC, as well (Figs. 3b–c). The two centromere-near probes RP11-172D7 and RP11-81B3 in 22q11.21 gave two co-localized signals, each (Figs. 3b–c), while the probe RP11-1058B20 in 22q11.22 was absent on the sSMC. In summary, a dic(13 or 21;22)(13 or 21pter->13 or21q11::22q11.1~11.2->22q11.21~11.22::22q11.21~11.22->22pter) was characterized.

### Discussion

Here we report three new cases of patients with unique complex sSMC. Two of the sSMC are maternally derived (cases A and B) and one is *de novo *(case C). The two maternally derived sSMC both lead to partial trisomies of the short arm of chromosome 18 and interestingly two similar cases are already reported in the literature [8,9] (cases 2 and 3 in Tab. 1). Compared to cases with partial tetrasomy of the short arm of chromosome 18 (overview on 140 cases reported in the literature see [10]), those four cases with partial trisomy 18p present with surprisingly mild clinical signs and symptoms.

The third case reported here (Case C) is a child with a cat eye syndrome which is carrier of a dicentric sSMC leading to a partial tetrasomy of 22q11.21. However, it is the first cat eye syndrome associated sSMC with centromeres derived from two different chromosomes, i.e. 13 or 21 and 14 or 22, even though 132 cases are already reported [10]. It is suggested that acrocentric derived dicentric inverted duplicated sSMC are formed due to an U-type exchange during meiosis [1]. Thus, the most likely explanation for this unusual sSMC in case C is, that one of the original chromosomes 22 already had a polymorphic centromeric region with D13/21Z1 instead of D14/22Z1 sequences in its centromeric region. A similar polymorphic behavior for the sequence D15Z1 was recently reported to be present in 17.6% of the acrocentric chromosomes [11]. An alike mode of formation could be suggested for cases 4, 6, 12, 14 and 15 of Tab. 1.

**Figure 4:**
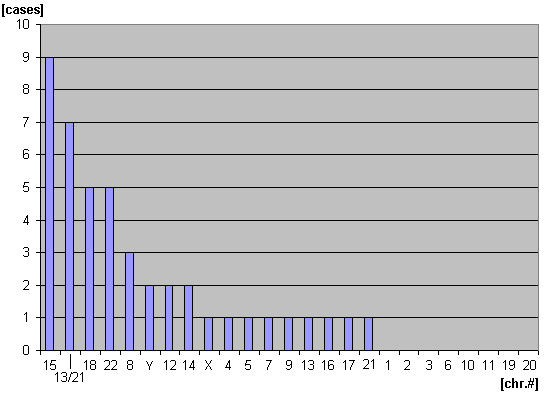
Involvement of the 24 different human chromosomes into the formation of unique complex sSMC.

Overall, now 22 complex sSMC are reported in the literature (see Tab. 1). According to Fig. 4 it can be reckoned that in principle all chromosomes can be involved in their formation; examples for chromosomes #1, #2, #3, #6, #10, #11, #19 and #20 will be detected, when more unique complex sSMC are reported. Nonetheless, Fig. 4 also indicates, that some chromosomes might be involved more often in complex sSMC than others, i.e. #13/21, #15, #18 and #22, possibly also #8. Due to low case number it can only be speculated if, similar to the formation of the der(22)t(11;22), specific DNA-sequences are causative for their formation [12]. At least this could be speculated for cases 13 and 18 from Tab. 1, where the breakpoint in chromosome 8 was 8p23.2. Here it is known that low copy repeats co-mediate recurrent rearrangements consisting of triplication at 8p23.2 [13].

**Figure 5:**
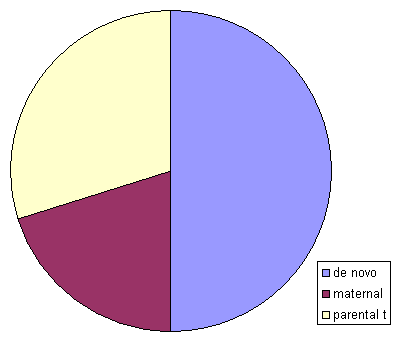
Origin of unique complex sSMC: *de novo*, maternal or due to a parental translocation (parental t).

According to Tab. 1 and summarized in Fig. 5 only 50% of unique complex sSMC are *de novo*. The remainder 50% are parentally derived: 20% of the patients inherited the sSMC directly – here only maternal inheritance is reported yet. In the remainder 30% of the cases the unique complex sSMC was part of a balanced translocation in one parent. The latter resembles to the mode of formation of the most frequent complex sSMC, the der(22)t(11;22) [12].

**Figure 6:**
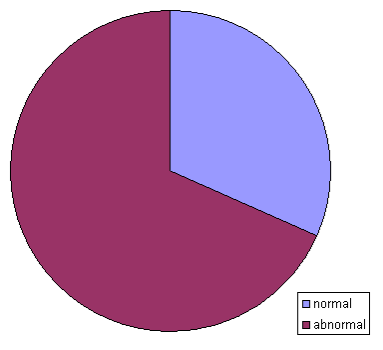
Clinical outcome of 19 of the 22 cases with a unique complex sSMC.

In 19/22 cases summarized in Tab. 1 the clinical outcome was reported. In ~1/3 of the cases a normal phenotype and in the rest an in parts severely abnormal clinical outcome was present (Fig. 6).

## Conclusion

Among ~2500 reported sSMC cases studied for their chromosomal origin and subsequently reported, by now 22 cases with unique complex sSMC were detected [10]. I.e. unique complex sSMC are to be expected in at least 0.9% of patients with an sSMC. However, the question is, if the percentage of this specific kind of sSMC is not underestimated. Unique complex sSMC are easy to be missed if, in case of acrocentric chromosome derived sSMC not all centromeric probes are applied, and/or if no flow sorting or microdissection followed by reverse FISH or array-CGH [14] is performed. Thus, for cases similar to cases 2, 3, 9, 10, 15 and 16 from table 1 one may suggest there is no euchromatin on the sSMC after its origin from an acrocentric chromosome was revealed by a centromeric probe. Or, as in case 13, if already a (relatively large) euchromatic imbalance was detected, which could explain the clinical symptoms of the specific patient, a very small part of another chromosomal origin is very unlikely to be detected. This can be problematic especially in prenatal diagnostics, but also concerning genotype-phenotype correlations of sSMC.

In conclusion, a really comprehensive characterization of all sSMC by different probes, probe sets and approaches could enhance the detection rate of unique complex sSMC. Unique complex sSMC are especially to be expected in cases with a 'heterochromatic sSMC', no uniparental disomy in connection with the sSMC and, nonetheless, clinical symptoms. Here a reverse FISH or array-CGH experiment of the sSMC should be performed and might show additional chromosomal imbalances.

## Methods

### Cytogenetics and molecular cytogenetics

Banding cytogenetics (GTG-banding and NOR-staining) was done on metaphase cells derived from peripheral blood of the three aforementioned patients and their parents according to standard procedures. 25 cells were analyses per case.

The sSMC were characterized in more detail by commercially available centromeric probes or centromere-specific multicolor fluorescence in situ hybridization (cenM-FISH) [15], subcentromere-near [16] (#13, #18, 21, #22) and commercially available subtelomeric FISH-probes (#8 and #18, Vysis) and/or home made partial chromosome painting probes for the long and the short arm of chromosome 18 [16] plus the short arm of all acrocentric chromosomes (probe midi54 [17]). Additionally, the multicolor banding (MCB) probe set for chromosome 18 [18] was applied in case A. In case C the probes RP11-172D7 and RP11-81B3 in 22q11.21 plus RP11-1058B20 in 22q11.22 were used to characterize the size of the cat eye syndrome specific tetrasomy. In case A and B also chromosome flow sorting [19] or glass needle based microdissection of the sSMC were done [2], respectively, followed by reverse FISH. All aforementioned molecular cytogenetic approaches are standard techniques of (multicolor) FISH and were repeatedly described before in detail.

### Review of the literature

The sSMC-related literature is collected from [10]. The database was searched for complex sSMC cases, which were included in Table 1.

## Competing interests

The author(s) declare that they have no competing interests.

## Authors' contributions

VT and MG performed chromosome flow sorting and FISH analysis; SF, AN and LR provided the clinical cases and description, FB, JA and LR performed banding cytogenetic analyses and detected the sSMC, MG, HM, EE and DR did molecular cytogenetic studies, NK performed microdissection and reverse FISH, AW, TL have been involved in drafting the manuscript and revising it critically for important intellectual content. All authors read and approved the final manuscript.
